# Perceptions of Fourth-Year Medical Students on Writing and Presenting Case Reports

**DOI:** 10.7759/cureus.2341

**Published:** 2018-03-19

**Authors:** Pinky Jha, Abhishek Thakur, Jennifer Klumb, Sanjay Bhandari

**Affiliations:** 1 Medicine, Medical College of Wisconsin; 2 School of Medicine, Frank H Netter Md School of Medicine; 3 Medicine-Education Programs, Medical College of Wisconsin

**Keywords:** medical students, case report writing, mentorship, barriers

## Abstract

Background

Writing and presenting case reports has educational benefits for medical students by providing them a platform for involvement in scholarly activities and promoting scientific writing and critical thinking. This study assesses medical students’ perceived benefits, challenges, and barriers regarding the process of writing and presenting case reports.

Methods

A qualtrics survey was emailed to 225 fourth-year medical students of the Medical College of Wisconsin. All of the questions in the survey were focused on obtaining the students’ perceptions of factors facilitating the writing or presenting of case reports, its benefits, and the perceived challenges and barriers to writing and presenting case reports. Responses were obtained on a five-point Likert scale. We performed conventional quantitative analysis on all the responses and analyzed the data as respective proportions. A comparison between the responses of those who have and have not presented case reports previously was performed using a chi-square test or Fischer exact test.

Results

Of the 84 medical students who completed the survey, 67% said they have not written or presented case reports in regional or national meetings. Ninety-nine percent of the total students believed (‘agreed’ or ‘strongly agreed’) that finding a good mentor was an important factor in facilitating the process of writing and presenting case reports, and 98% perceived finding an interesting case as equally important. The top three perceived benefits of writing or presenting case reports included improving scientific-writing skills (95%), enhancing curriculum vitae (CV) and securing residency positions (93%), and improving presentation skills (90%). The main perceived barriers to writing or presenting case reports included lack of formal training (74%) and lack of a mentor (71%). In a subgroup analysis, 95% of students who have never previously written or presented a case report, versus 79% of students who have done so, perceived that a lack of formal training and/or a lack of a mentor were major barriers to writing or presenting case reports (p=0.054).

Conclusion

Our study shows that the majority of M4s reported that they have not written or presented any case reports and that writing case reports has multiple educational values with considerable barriers. Our findings highlight the importance of good mentorship and formal training in case report writing.

## Introduction

A case report is a document that generally describes one or more patient encounters, which may be novel or unique.The goal of such a narrative includes the sharing of information for medical, scientific, or educational purposes [1]. Individual case studies generally do not have great strength of evidence or generalizability and therefore, do not contribute significantly to evidence-based medicine [2]. However, they can play an important role in the knowledge and education of medical students, residents, and fellows.

The process of planning, writing, and presenting case reports provides medical students the opportunity to engage in scholarly activities and to develop their academic research skills. Additionally, students develop scientific-writing and critical thinking skills, while also appreciating the processes of publication and professional development. Medical schools can assist students in expanding these skills by supporting them in the process of developing and presenting scientific literature, potentially through curricular integration and greater resource availability [3].

Although case reporting can be an effective teaching tool with a broad range of potential educational benefits, there is limited knowledge on the perceptions of medical students regarding case report writing. In this study, we surveyed medical students to assess their perceived benefits, challenges, and barriers regarding the process of writing and presenting case reports.

## Materials and methods

A qualtrics survey was emailed to all the fourth-year medical students (M4s), and the responses were followed for over a week, after which the survey was closed. Responses were obtained on five-point Likert scales (‘strongly disagree’, ‘disagree’, ‘undecided’, ’agree’, and ’strongly agree’). An initial pilot survey-based study was done to design the survey questionnaire for the present study. The pilot study was conducted on a few students who had previous experience in case report writing and presenting. The participants were asked open-ended questions related to writing or presenting case reports. We incorporated the themes generated from the respondents’ narratives to design the questionnaire for this study.

All the questions in the survey were focused on obtaining the students’ perceptions on factors facilitating writing or presenting case reports, its benefits, the perceived challenges, and barriers to writing and presenting case reports (Table 1).

We performed quantitative analysis of all the responses. All responses were analyzed as respective percentages. For convenient interpretation, responses pertaining to ‘strongly agree’ and ‘agree’ were categorized as the positively-perceived responses, and the remaining responses as negatively-perceived responses. When needed, the comparison between the responses of those who have and have not written or presented case reports previously was performed using a chi-square test or Fischer exact test. All analysis was performed using SAS 9.4 (Cary, NC). P<0.05 was considered statistically significant.

The study was approved by the institutional review board (IRB) of the Medical College of Wisconsin.

## Results

**Figure 1:**
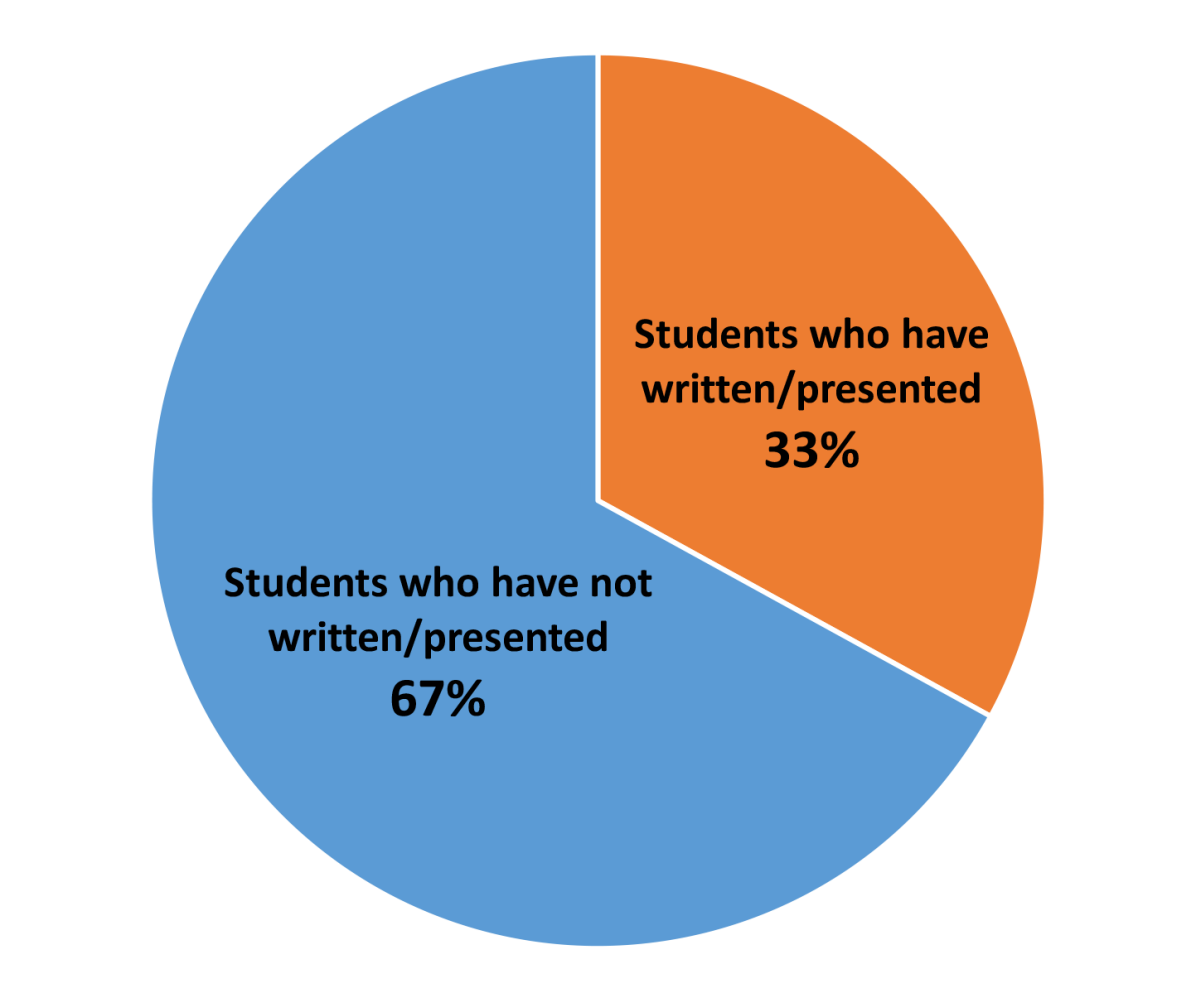
Pie chart showing the survey results performed on fourth year medical students based on whether they have written/presented case reports in any regional or national meetings.

The survey questionnaire was sent to a total of 225 M4s. Out of them, 91 responded (response rate of 40%). Seven were excluded since they did not complete the survey. The total students included in the study were 84, of which 56 students (67%) reported that they have not written or presented case reports in any regional or national meetings (Figure 1).

Ninety-nine percent of the students believed (‘agreed’ or ‘strongly agreed’) or positively perceived that finding a good mentor was an important factor in facilitating writing and presenting case reports. Finding an interesting case was perceived equally positively (98% responses as ‘agreed’ or ‘strongly agreed’). Improving scientific-writing skills (95% responses as ‘agreed’ or ‘strongly agreed’), enhancing curriculum vitae (CV) and securing residency positions (93%), and improving presentation skills (90%) were the top three benefits of writing and presenting case reports as perceived by the students. Lack of formal training in writing case reports (74% responses as ‘agreed’ or ‘strongly agreed’) and lack of a mentor (71%) were the major reported barriers to writing or presenting case reports (Table 1).

**Table 1:** Survey questions with respective medical students’ responses.

Survey Questions	Agree/Strongly Agree (N=84)
Factors that facilitate writing and presenting case reports:	
Finding a good mentor	99%
Finding an interesting case	98%
Having financial assistance	73%
Lectures and workshops	65%
Benefits of writing and presenting case reports:	
Improves scientific-writing skills	95%
Enhances CV and secures residency positions	93%
Improves presentation skills	90%
Improves critical thinking	82%
Networking and collaboration	82%
Barriers to writing and presenting case reports:	
Lack of formal training in writing case reports	74%
Lack of mentor	71%
Lack of time in medical school	66%
Lack of training in reviewing scientific literature	57%
Lack of financial assistance	54%
Lack of opportunities/venues to present	46%

**Figure 2:**
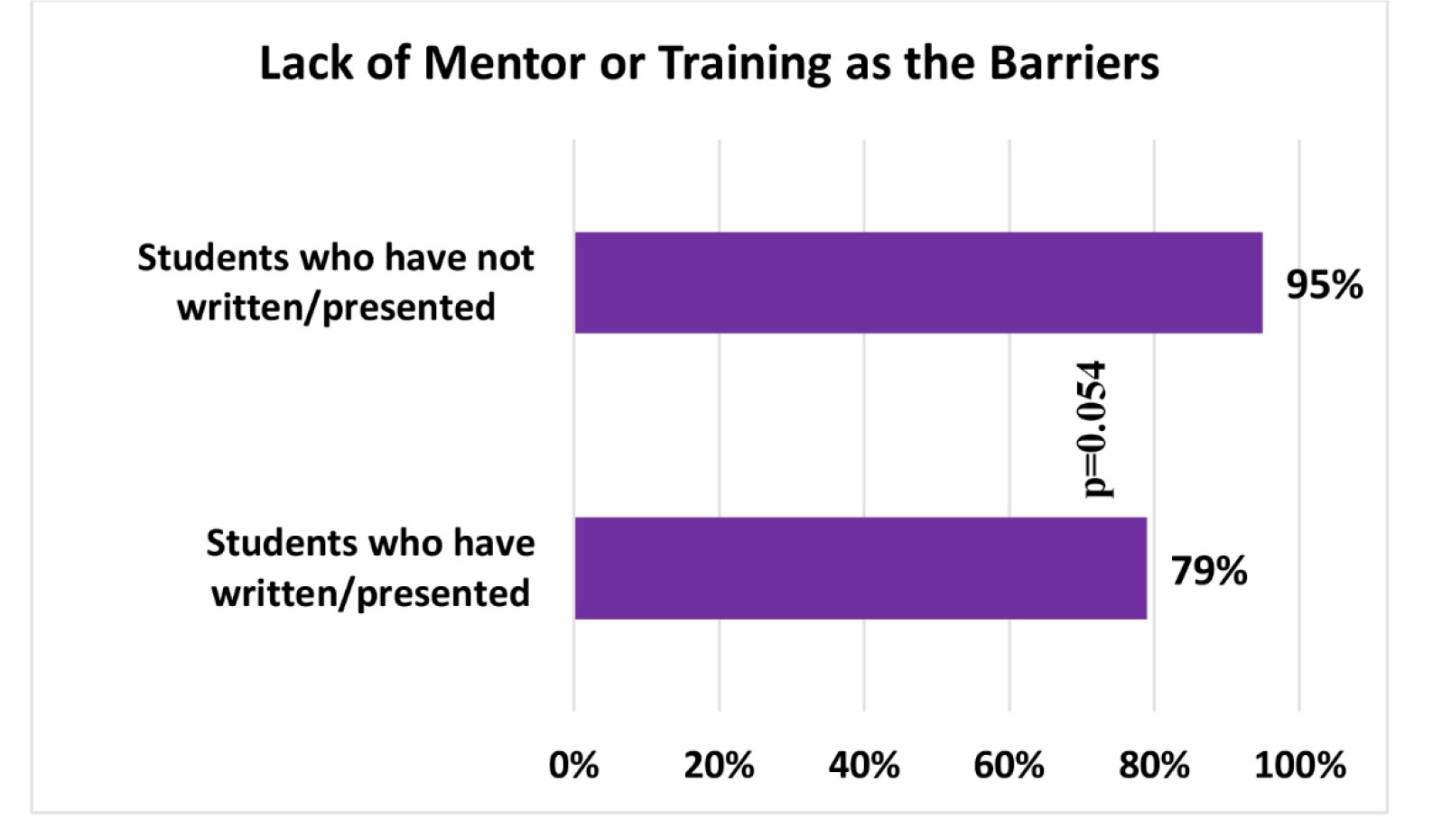
Bar chart showing lack of mentor/formal training as the major barriers to writing/presenting case reports as perceived (agreed or strongly agreed) by the students who have written/presented case reports vs. those who have not.

In a subgroup analysis, 95% of the students who have never written or presented any case reports, versus 79% of the students who have done so, perceived that a lack of formal training in writing case reports and/or the lack of a mentor were the major barriers to writing or presenting case reports (p=0.054) (Figure 2).

## Discussion

While case reports are graded as lower down the hierarchy of level of evidence, they help illustrate the unusual presentation of common diseases, presentation of uncommon diseases, and even lead to the recognition of new diseases. They can also raise a myriad of unanswered questions that may open doors to generating hypotheses for future research [3-5]. In a study that analyzed case reports and case series from a high-impact journal, 24 out of a total 103 case reports/case series were actually followed by randomized clinical trials (RCTs) [6]. It is this platform of meticulous case reporting that led to the discovery of Cushing’s disease by Harvey Cushing [7], and our present understanding of the association of thalidomide and congenital disease [8]. In addition to providing insight into diseases, case reporting offers substantial educational benefits, especially for students. This includes cultivating critical thinking, making use of opportunities to collaborate with faculty mentors, familiarizing with the peer review process, writing and publishing [9-11]. Writing case reports can help students sharpen their observational skills, generate hypotheses, appreciate patient-centered care, and motivate them to become involved in scholarly activities [4].

As clinician educators, we agree with Packer et al. about the value of the educational benefits resulting from student-generated case reports [3-4]. Our survey-based study performed on fourth-year medical students shows that students perceive benefits and barriers to writing case reports. Students see the professional value of case reporting as it adds to their CV or residency applications, and provides opportunities to attend conferences and network. They also gain skills in scientific-writing, and in the understanding of the purpose and process of case reports, clinical reasoning, and the preparation of posters and presentations. 

Despite the several benefits described, our study showed that the majority of M4s, which included more than two-thirds of them, said they have not presented case reports in regional or national meetings. The major barriers to writing and presenting case reports, as perceived by the students, included the lack of formal training in writing reports and a knowledgeable mentor, essentially one who can help identify an appropriate case with educational value, and assist with writing and submitting an abstract/case report. In a subgroup analysis, we found no significant difference in this perception between the students who have previously written or presented case reports, versus those who have not (p=0.054). It was not clear from our study if the students who have previously written or presented case reports would have been able to write and present more case reports had they been provided a formal training or had a good mentor. Previous studies have identified that educational interventions and institutional support for both students and faculty (such as training workshops and faculty development programs) can help create excellent role models (mentors) and reduce barriers in writing case reports [12]. Training can be in the form of workshops and educational sessions focused on providing assistance to the students on case report writing and presenting. 

One of the strengths of this survey is the acquisition of responses from both students with and without experience in writing case reports. The result reflects perspectives of all M4s and, thus, we were able to determine differences between both groups of students. As with other online questionnaires, this survey was cost-effective with rapid data acquisition [13-14]. Several limitations of this study should be considered. Our study did not assess whether the medical students were motivated to write case reports or if there were any factors which could help to stimulate their motivation. This can be an area of future studies. Our study presumed writing and presenting case reports (oral or poster) in various meetings as part of the same spectrum. Our study fails to highlight whether students perceive writing and presenting case reports as different entities with different perceived benefits and barriers. Further studies are needed to elucidate these possible differences. Ninety-three percent of M4s said that case report writing or presenting would help them build their CVs and secure residency positions. The various types of residencies M4s are seeking and whether their responses will differ depending on the types of residencies can be some of the interesting questions for a future study. The response rate of 40% is not optimal, and a larger sample size would provide a better understanding of students' perceptions surrounding case reporting. Web-based surveys tend to have higher nonresponse rates when compared to more traditional data collection modes such as paper-based surveys, though current studies reveal that this gap in coverage has been diminishing in more recent years [13-14]. Our study only focuses on M4s rather than third-year medical students (M3). We believe our study on M4s reflects the perceptions of medical students in a true sense since M4s typically constitute the last batch in a medical school and possess all the experiences they have acquired throughout the years in the school. Further, our study on M4s also comprises the perceptions they have on the completion of their first clinical year, in addition to their preparation for residency application. Nevertheless, a separate study on M3s can be an area of future research. Our study does not examine faculty perception. Although the lack of mentorship is identified as a barrier in our study, further research is necessary to assess perceptions of faculty members regarding case report writing in medical education. There is a need for more studies to review barriers for faculty members, and possible interventions to overcome the challenges. There are limited data regarding the role of case report writing as well as mentorship in writing or presenting case reports in medical education and, therefore, more research is needed in the area. Additionally, well-documented limitations pertaining to most web-based surveys should be considered here including a potential decrease in validity and generalizability [13-14]. The results of this study reflect the perception of the students at the Medical College of Wisconsin only, and future comparative studies may be necessary for data to be generalized to other medical schools.

The structure of medical education in the United States has changed drastically in recent decades, now incorporating more problem-based learning, clinical reasoning, and case-based sessions. However, even recent studies continue to stress the need for dedicating additional resources to develop a clinical reasoning curriculum throughout the four years of medical school [15]. The Accreditation Council for Graduate Medical Education (ACGME) encourages the adoption of such teaching methodologies in self-directed learning, and thus, many residents are now required to complete scholarly activity during their training [3, 16]. However, such requirement does not exist for medical students despite the benefits. According to a Liasion Committee on Medical Eeducation (LCME) survey in 2016-2017, only 62 out of 145 participating medical schools or about 43% of them have a research requirement for medical students [17]. As demonstrated by this survey, incorporating scientific-writing and presentation can be an integral and beneficial part of their curriculum.

## Conclusions

In conclusion, our survey-based study shows that the majority of medical students have not been involved in writing and presenting case reports and the potential lack of necessary resources may be a major contributing factor. Our findings support the need to develop curricula in medical schools and mentorship programs to address the systematic and effective teaching of medical writing. Further, additional institutional resources should be dedicated to design a curriculum that promotes clinical case-based scholarly activity and mentorship earlier in medical school.
